# Herpes Simplex Virus 1 Infection of Neuronal and Non-Neuronal Cells Elicits Specific Innate Immune Responses and Immune Evasion Mechanisms

**DOI:** 10.3389/fimmu.2021.644664

**Published:** 2021-05-31

**Authors:** Amanda L. Verzosa, Lea A. McGeever, Shun-Je Bhark, Tracie Delgado, Nicole Salazar, Erica L. Sanchez

**Affiliations:** ^1^ Biology Department, College of Science and Engineering, San Francisco State University, San Francisco, CA, United States; ^2^ Biology Department, Seattle Pacific University, Seattle, WA, United States

**Keywords:** alphaherpesvirus, HSV-1, innate immunity, neuronal, latency, TLR - toll-like receptor, cGAS-STING pathway, IFN - interferon

## Abstract

Alphaherpesviruses (α-HV) are a large family of double-stranded DNA viruses which cause many human and animal diseases. There are three human α-HVs: Herpes Simplex Viruses (HSV-1 and HSV-2) and Varicella Zoster Virus (VZV). All α-HV have evolved multiple strategies to suppress or exploit host cell innate immune signaling pathways to aid in their infections. All α-HVs initially infect epithelial cells (primary site of infection), and later spread to infect innervating sensory neurons. As with all herpesviruses, α-HVs have both a lytic (productive) and latent (dormant) stage of infection. During the lytic stage, the virus rapidly replicates in epithelial cells before it is cleared by the immune system. In contrast, latent infection in host neurons is a life-long infection. Upon infection of mucosal epithelial cells, herpesviruses immediately employ a variety of cellular mechanisms to evade host detection during active replication. Next, infectious viral progeny bud from infected cells and fuse to neuronal axonal terminals. Here, the nucleocapsid is transported *via* sensory neuron axons to the ganglion cell body, where latency is established until viral reactivation. This review will primarily focus on how HSV-1 induces various innate immune responses, including host cell recognition of viral constituents by pattern-recognition receptors (PRRs), induction of IFN-mediated immune responses involving toll-like receptor (TLR) signaling pathways, and cyclic GMP‐AMP synthase stimulator of interferon genes (cGAS-STING). This review focuses on these pathways along with other mechanisms including autophagy and the complement system. We will summarize and discuss recent evidence which has revealed how HSV-1 is able to manipulate and evade host antiviral innate immune responses both in neuronal (sensory neurons of the trigeminal ganglia) and non-neuronal (epithelial) cells. Understanding the innate immune response mechanisms triggered by HSV-1 infection, and the mechanisms of innate immune evasion, will impact the development of future therapeutic treatments.

## Introduction

### Herpesviruses

The *Herpesviridae* family is a large family of viruses that infects both humans and animals. Herpesviridae is derived from the Greek “*herpein*” meaning “*to creep*” (1). Structurally, herpesviruses contain four layers. First, the herpesvirus *genome* consists of linear double-stranded DNA (dsDNA), ranging in size between ~120-250 kilobases (2, 3). Second, the viral DNA genome is enclosed by a protein icosahedral *capsid*, approximately 100 to 110 nanometers in diameter (4). Third, *tegument* proteins, an amorphous viral protein matrix of 30 or more proteins, surrounds the capsid and is poorly defined (5). Fourth, herpesviruses are encapsulated by a lipid *envelope* which contains both viral glycoproteins and some host cellular proteins (6, 7).

The Herpesviridae family consists of eight types of human herpesviruses (HHVs), belonging to three subfamilies: Alphaherpesvirinae (α–HV), Betaherpesvirinae (β–HV) and Gammaherpesvirinae (γ–HV) (8). Their characteristics are summarized in **Table 1**.

**Table 1:** Human Herpesviruses.

HHV	Virus Name	Subfamily	Abbreviation(s)
HHV–1	Herpes simplex–1 virus	α	HHV–1/HSV–1
HHV–2	Herpes simplex–2 virus	α	HHV–2/HSV–2
HHV–3	Varicella zoster virus	α	HHV–3/VZV
HHV–4	Epstein–Barr virus	γ	HHV–4/EBV
HHV–5	Cytomegalovirus	β	HHV–5/CMV
HHV–6	N/A	β	HHV–6
HHV–7	N/A	β	HHV–7
HHV–8	Kaposi’s Sarcoma Herpesvirus	γ	HHV–8/KSHV

Each herpesvirus is classified based on their biological characteristics and tissue tropism during primary (lytic) and latent infections (8). α–HV lytic infections have a short reproductive cycle, leading to rapid destruction of infected host cells. While α–HV’s have a broad host range, they primarily infect mucosal epithelial cells during initial infection and neuronal ganglia during latent infection (9). β–HV lytic infections have a relatively longer reproductive cycle, with a large host range and the ability to latently persist in monocytes or hematopoietic stem cells (10). γ–HVs have a variable reproductive cycle length and a narrow host range, which is restricted to the family or order to which the natural host belongs (8, 11). γ–HVs traditionally establish latent infection in lymphoid tissues and are associated with lymphoproliferative diseases (8).

**Figure 1:**
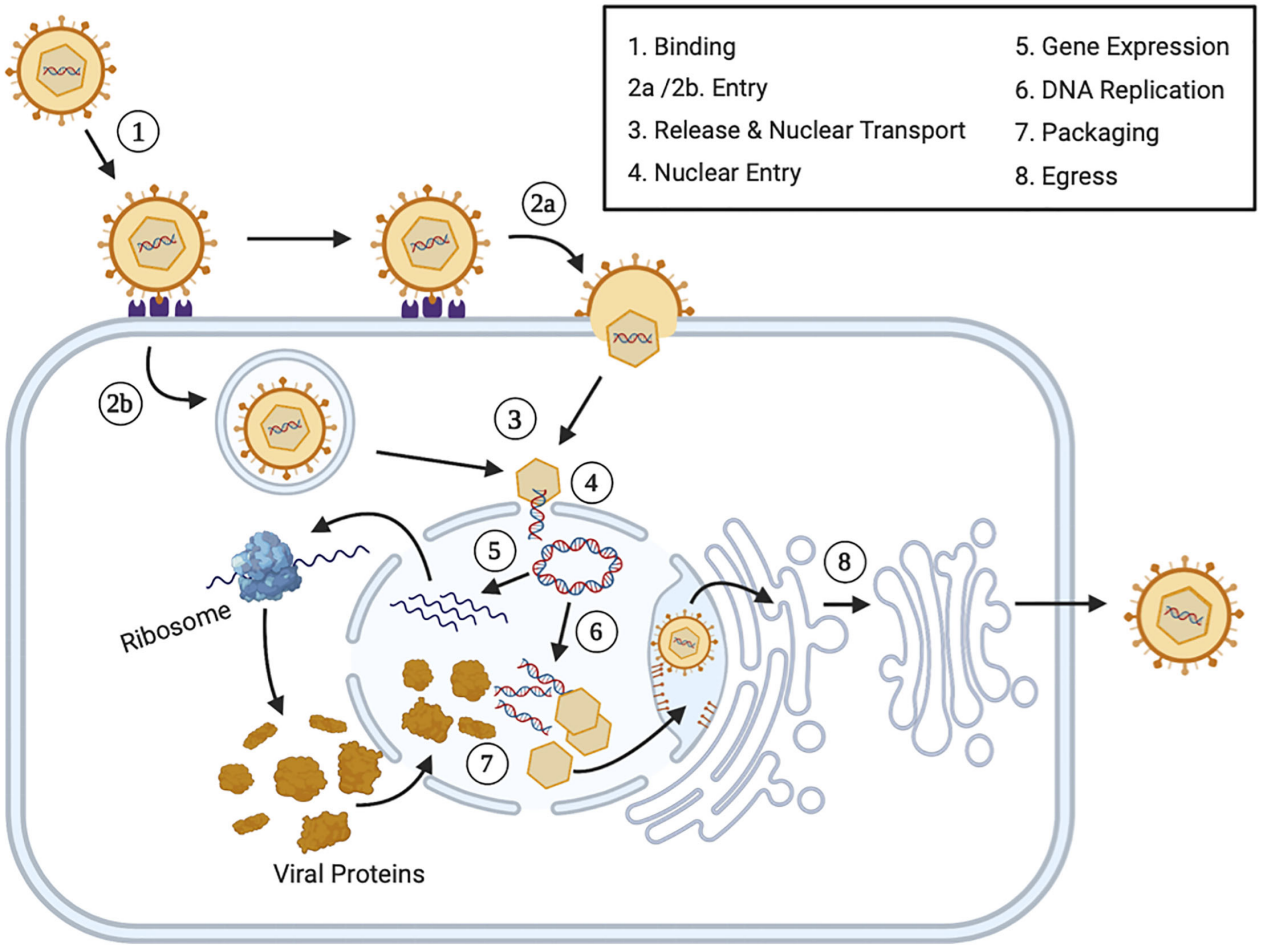
The Lytic Human Herpesvirus Life Cycle. Step 1 (*Binding*): During primary lytic infection, HHVs bind extracellular host cells receptors using specific envelope viral glycoproteins. Step 2 (*Entry*): HHVs enters the cell *via* fusion through receptor mediated endocytosis (2a) or endosome formation (2b). Step 3 (*Release and Nuclear Transport*): After viral uncoating, both the nucleocapsid and tegument proteins are released into the cytoplasm. The nucleocapsids are transported *via* cytoskeletal structures or diffusion to the nucleus. Step 4 (*Nuclear Entry*): The viral genome plus some associated viral proteins, including some tegument proteins, enter the nucleus *via* nuclear pores and the viral genome circularizes. Step 5 (*Gene Expression*): Immediately early (IE) viral genes, early (E) viral genes and late (L) viral genes are expressed in a temporal fashion. Each set of mRNAs are transported to the cytoplasm and translated into protein before returning to the nucleus and before initiating the next set of viral genes. Step 6 (*DNA Replication*): Early viral gene expression initiates viral DNA replication. Step 7 (*Packaging*): Late viral structural proteins assemble into viral capsids and they are packaged with DNA. Step 8 (*Egress*): Viral progeny bud through the inner nuclear membrane and enter the intermembrane space. Virions are transported to the nuclear associated endoplasmic reticulum and are transported to the cellular plasma membrane, where they are released *via* cell fusion, exocytosis or cellular lysis.

**Figure 2:**
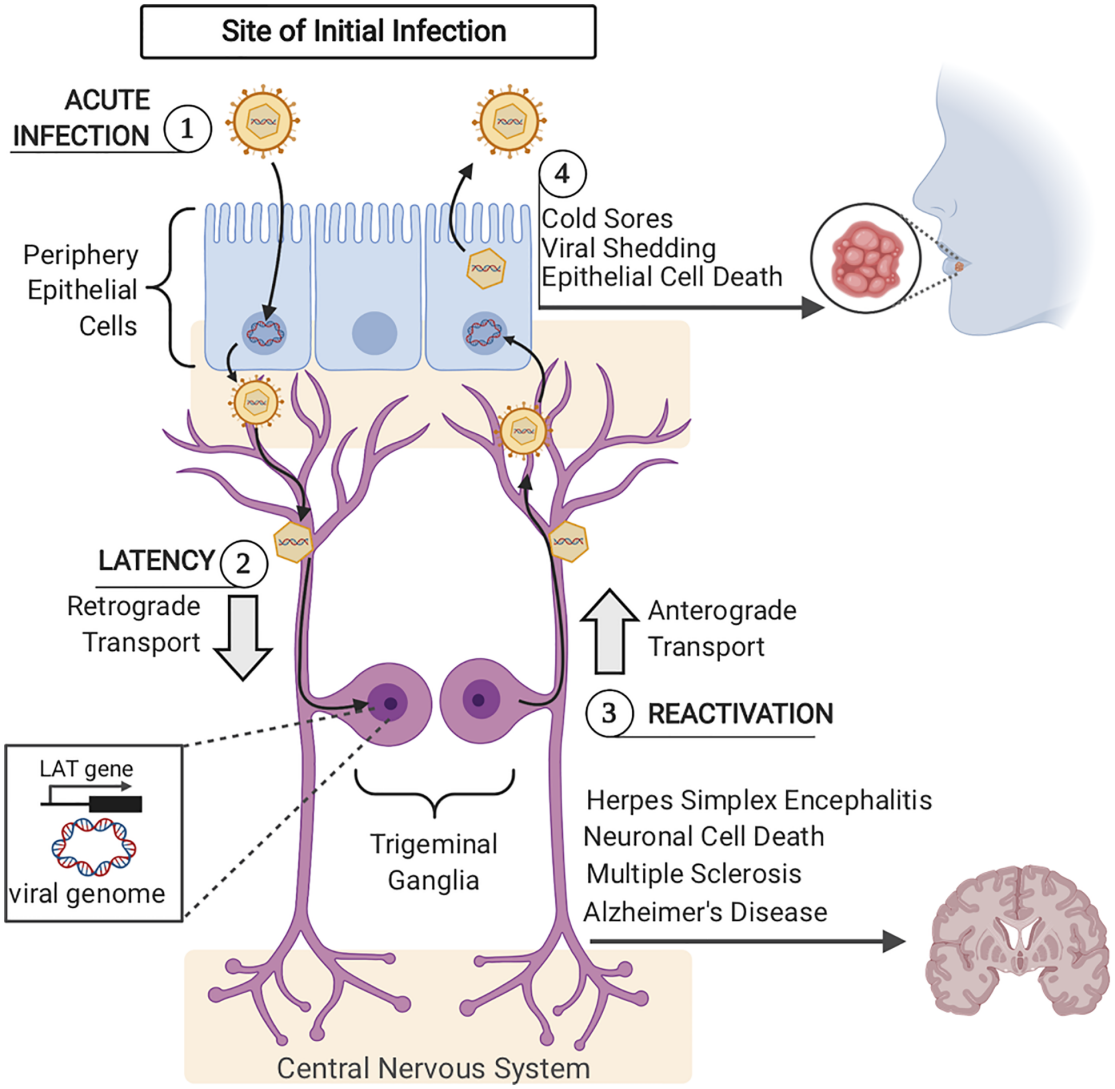
Acute and Latent HSV–1 Infection (1). Acute HSV–1 infection is initiated when infectious virions enter epithelial cells *via* viral envelope fusion with the plasma membrane. The viral nucleocapsid reaches the epithelial cells nucleus and the viral genome enters. In the nucleus, viral genome replication and viral gene expression occur to produce more infectious virions. Newly formed viral particles are released, some of which infect nearby innervating sensory neurons. (2) Via retrograde trafficking, HSV–1 capsids reach the neuronal cell body in the sensory ganglia (trigeminal ganglia). In the neuronal nucleus, the viral DNA circularizes, causing the host cell to silence viral genome transcription, except for the latency– associated transcript (LAT) gene. If viral progeny reach the central nervous system, this can lead to herpes simplex encephalitis, neuronal cell death, and has more recently been connected to long–term pathogenesis including Multiple Sclerosis and Alzheimer’s Disease. (3) Upon viral reactivation, viral nucleocapsids leave the neuronal nucleus and travel back to epithelial cells *via* anterograde trafficking. (4) Once virions arrive at the epithelial cells, viral replication is once again initiated, viral progeny are assembled and released, causing epithelial cell death and orofacial sores.

Herpesviruses exhibit both lytic (productive) and latent (dormant) infection life cycles (1, 12). During primary lytic herpesvirus infection, the virus replicates and produces new viral progeny in host cells, often resulting in cellular death (**Figure 1**). During primary lytic infection, there is symptomatic and asymptomatic shedding of virus. Once the host immune response is elicited, HHVs characteristically establish latency and hide in secondary host cells in order to prevent detection by the immune system (13). During latency, the viral DNA can either integrate to the host genome or tether to host DNA as a circular episome, and expresses very few viral genes (14–16). The virus can persist in the latent form forever. Periodically, the virus can reactivate from latency due to various related host factors (17). During reactivation, the virus typically returns to the primary site of infection and undergoes lytic replication until the host immune response forces it back into latency (18) (**Figure 2**).

### Human Herpesviruses: Clinical Manifestations and Epidemiology

#### Alphaherpesviruses

Human Herpesviruses cause a wide variety of diseases, which are most often manifested during primary lytic infection. Herpes Simplex Virus 1 (HSV–1) and Herpes Simplex Virus 2 (HSV–2) cause primary infections in epithelial cells and establish latency in neuronal ganglia (9, 12). Both HSV–1 and HSV–2 infections are widespread among humans globally and clinically manifest as skin ulcerations and flu–like discomfort in infected individuals. HSV–1 infection is primarily transmitted by oral–to–oral contact and commonly causes oral cold sores (19). HSV–1 can also be transmitted sexually *via* oral–to–genital contact and subsequently cause genital sores. HSV–1 is a life–long and persistent infection with ~55% of the US population infected in 2018 (20). In approximately 1 in 250,000 to 1 in 500,000 individuals per year, HSV–1 reactivates backwards towards the brain, and causes herpes simplex encephalitis, leading to inflammation, necrosis, and liquefaction of brain tissue (21). Children and adolescents account for approximately one third of all cases and result in a greater than 70% mortality rate. In 10 out of every 100,000 births globally, infants exposed to HSV–1 or HSV–2 in the genital tract during delivery develop neonatal herpes, which results in severe neurological disability or death (22). The risk of neonatal herpes transmission is highest when the mother is infected for the first time during her pregnancy (23). HSV–2 infection is almost entirely sexually transmitted and causes genital sores (24). In 2015, approximately 10–20% of people aged 18–49 in the USA were infected with HSV–2 (25). HSV–1 and HSV–2 have a greater transmission rate when there are active sores present (26). However, most infections are asymptomatic despite active shedding of viruses, leading to undetected infections and spread (26).

Varicella–Zoster Virus (VZV) causes varicella (chickenpox) during primary infection of epithelial cells and establishes latency in neuronal dorsal root ganglia. The clinical manifestations of VZV infection includes skin rash, blisters, fever, pain, sore throat, and headache (27). VZV typically infects children but can infect people at any age and is transmitted by droplets. Children infected with VZV typically have minor symptoms, while adults have more severe symptoms (28). VZV’s reactivation from latency, and subsequent transport down sensory neurons, causes herpesvirus zoster (shingles): a very painful rash (29). Risk of shingles increases as an individual gets older, with almost 1 out of 3 people in the United States developing shingles during their lifetime (30). Before the VZV vaccine was introduced in 1995, greater than 95% of individuals were naturally infected with VZV by adulthood (31). However, since vaccination, the primary VZV disease incidence has been reduced by 80–90% (32).

#### Betaherpesviruses

Cytomegalovirus (CMV) infection causes “mononucleosis–like syndrome” (fever, rash, sore throat, nausea, muscle aches, swollen glands, and fatigue) during primary infection (12). CMV infection is a significant cause of congenital disease. In mothers, first–time infection or latent CMV reactivation during pregnancy, particularly during their first trimester, can lead to congenital defects, mental retardation, hearing and vision loss in their infants (33). While most people are infected with CMV at some point during their lifetime, it typically results in no symptoms. Immunocompromised patients are most susceptible to CMV and often have severe and life threatening outcomes (34, 35). Estimates of seroprevalence of CMV in the US ranges from 40% to 83%, with lower socioeconomic status correlating with higher infection rates (36). CMV primarily infects epithelial cells of the respiratory tract, salivary glands, and kidneys and undergoes latency in monocytes or hematopoietic stem cells (37, 38). CMV is primarily transmitted by saliva and urine (39, 40).

HHV–6 and HHV–7 are the least characterized human herpesviruses. HHV–6 and HHV–7 typically infect children during their early years of life (12). Primary HHV–6 and HHV–7 infections are associated with roseola (exanthem subitum) and fever, with most infections being minor or asymptomatic. HHV–7 infection is less virulent than HHV–6, with HHV–7 rarely causing symptomatic disease. Both HHV–6 and HHV–7 have universal prevalence in persons 6 years old and older (41). Both HHV–6 and HHV–7 infect T–lymphocytes, with the latent infection target site and mechanism of spread still under investigation (42, 43).

#### Gammaherpesviruses

Epstein–Barr Virus (EBV) causes infectious mononucleosis and is associated with Burkitt’s lymphoma. Primary EBV infection can cause fever, rash, sore throat, nausea, muscle aches, pain, swollen lymph nodes, fatigue, weight loss, and vomiting (44). Over 90% of the human population is infected with EBV, resulting in 200,000 cancer cases a year (45). EBV primarily infects and replicates in epithelial cells of the oropharynx and parotid gland and establishes latency in lymphocytes (46–49).

Kaposi’s Sarcoma Herpesvirus (KSHV) causes Kaposi’s Sarcoma, an endothelial cell derived vascular tumor that is common in acquired immunodeficiency (AIDS) patients and organ transplant recipients (50). KSHV is also associated with two B cell lymphoproliferative diseases, primary effusion lymphoma (PEL) and Multicentric Castleman’s disease (MCD). KSHV infections cause Kaposi’s Sarcoma (KS) in 1 out of every 200 transplant patients in the US (51). In the US, KSHV seroprevalence is estimated to be less than 10% and incidences of KS are usually below 0.1% (41). The primary modes of KSHV transmission include saliva, seminal fluid, nasal secretions, transplant of infected organs and blood transfusions (52).

### Alphaherpesvirus Infection of Neuronal and Non–Neuronal Cells

All alphaherpesviruses (α–HVs) cause primary infection in epithelial cells and establish latency in neuronal ganglia (12). Upon reactivation, HSV–1 and HSV–2 virions travel back to oral or genital epithelial cells where a new stage of productive infection initiates cutaneous and/or mucosal lesions. Both primary HSV infection, as well as reactivation events can lead to infection of the central nervous system (CNS) (53).

HSV–1 infection involves multiple cell types throughout the life–cycle of the virus. This review will primarily compare and contrast the immune response elicited by HSV–1 infected epithelial cells (non–neuronal) and sensory neurons, while also reviewing immune evasion mechanisms used by the virus at these same sites. HSV–1 infection is most often initiated *via* an orofacial route, entering the mucosal epithelium of the mouth, nose, or eyes (8). Upon infection, HSV–1 establishes lytic infection and undergoes multiple rounds of viral replication in epithelial cells. Infectious virions released from epithelial cells gain access to innervating sensory neurons, entering at axonal termini. HSV–1 virions traffic in a retrograde manner along neuronal axons to reach neuronal cell bodies in trigeminal ganglia (TG). While acute infection of epithelial cells will be cleared, virions that migrate to the cell bodies of sensory neurons and establish latent infections for the life of the host. Virions that reach neuronal nuclei enter the latency stage of infection, characterized by viral DNA circularization and episomal genome formation, resulting in limited expression of HSV–1 genes. Of the over 70 genes encoded by the HSV–1 genome, the non–coding latency–associated transcript (LAT) is the only viral RNA transcript highly expressed during HSV–1 latent infection in human dorsal root ganglia (54–56). HSV–1 LAT represses lytic gene expression and suppresses virus reactivation from latently infected neurons (55). LAT derived viral miRNAs have been shown to silence the expression of viral genes and prevent productive infection. Nonetheless, as with all herpesviruses, all latently infected cells hold the potential to reactivate to lytic replication and produce infectious virus (57). Upon reactivation, infectious virions are produced, which travel to axonal termini *via* anterograde trafficking to return to the initial site of infection. Subsequently, skin epithelial cells are infected and productive infection is established again, often resulting in epithelial cell death and the formation of recurrent blisters. Virus shedding during these reactivation events is a critical step in viral spread to new hosts.

Evidence has shown that the host immune response mounted during acute infection of epithelial cells is quite different from the immunological response at neuronal sites of infection. α–HV infections become latent in collaboration with immune suppression mechanisms. To evade host innate responses, HSV–1 has developed multiple mechanisms that attenuate host antiviral elements and facilitate its infection.

### Innate Immune Response and Immune Evasion

#### Overview of Innate Immunity and Mechanisms Herpesviruses Evade Effectively

Herpesviruses persist in human hosts by hiding from immune responses, which involve both innate and adaptive immune mechanisms. In this review, we focus on innate antiviral responses as they determine the outcome of viral load before the adaptive immune response can be activated. In the *immediate* response to infection, resident macrophages which are present in tissues without infection, represent the first line of defense against invading pathogens, while during active HSV–1 replication, macrophages can continue to infiltrate the TG (58, 59). In the *induced* innate response to infection, neutrophils are the first white blood cells recruited to sites of inflammation or areas of viral infection. Although a recent study of herpesvirus infection that caused neuroinflammation demonstrated neutrophils were not induced (60). The phagocytic process in neutrophils and macrophages is initiated through recognition of opsonized microbes by Fc receptors or complement receptors expressed on these phagocytes. Macrophages can engulf HSV–1 infected cells, and ubiquitinate the HSV–1 capsid to degrade it in a proteasome–dependent manner to expose the viral DNA to cytosolic DNA sensors and induce innate responses such as IFNβ (61). Here, we introduce innate immune mechanisms which herpesviruses evade effectively and we describe each of these processes with detailed examples from recent literature.

#### Interferon Response

The innate immune response to viral infection primarily consists of the induction of type I interferons (IFN–α and IFN–β). Interferons are a subgroup of cytokines released by host cells in response to viruses (and some bacteria) to help regulate the activity of the immune system. Interferons interfere with the propagation of viruses by producing proteins from IFN stimulated genes (ISGs) that create an antiviral state in infected cells and cells nearby (62). Release of IFN–α and IFN–β can induce an antiviral response by inducing IFN–responsive genes on neighboring cells that bind to the IFNα/β receptor and activate the JAK–STAT pathway to inhibit viral replication.

#### Plasmacytoid Dendritic Cells and Natural Killer Cells

Plasmacytoid dendritic cells (pDCs) and Natural Killer (NK) cells contribute to the innate immune response against HSV. pDCs can detect herpesvirus DNA in endosomes *via* Toll–Like–Receptors 9 and secrete massive amounts of type I interferon to prevent systemic spread of infection (61, 62). Interferon binding to receptors on circulating NK cells activate the NK cells to kill virus–infected cells (63). Yet, NK cells do not only depend on IFN to mediate anti–HSV immunity, as evidenced by patients that have functional IFN production, but absence of NK cell function, that are unable to clear severe HSV infections (62).

#### Toll–Like–Receptors (TLRs)

Toll–Like–Receptors are pattern recognition receptors (PRRs) that do not promote phagocytosis, but rather initiate intracellular signaling cascades that activate various cellular responses. TLRs recognize PAMPs from bacteria, fungi, and viruses. TLRs are present either on the plasma membrane or on endosomal membranes (64). Two major PRR families activate innate immunity in the central nervous system (CNS): the Toll–Like–Receptors (TLRs) and the Nod–like–receptors (NLRs). Since the first discovery report of a TLR4 in 1998, 10 human TLRs have been identified. TLRs are expressed in intracellular endosomal compartments (TLR3, TLR7, TLR8 and TLR9) or as transmembrane cell–surface receptors (all other TLRs). TLR3 activation increases type I IFN from microglia and monocyte–derived macrophages. TLR7 and TLR8 activation in CNS macrophages triggers canonical TLR signaling, leading to inflammation *via* NFkB and inflammatory cytokine production, including pro–IL–1β, which can trigger neuron death (65).

#### Cyclic GMP‐AMP Synthase Stimulator of Interferon Genes (cGAS–STING)

The cyclic GMP‐AMP synthase stimulator of interferon genes (cGAS–STING) is a cytosolic DNA sensor involved in the innate response to infection. cGAS generates cyclic dinucleotides (CDNs), including cGAMP that bind STING, leading to the activation of IFN regulatory factor 3 (IRF3) and resulting in IFN–β production (66).

#### The Complement System

Complement was initially established as the necessary blood serum component that completed antibody–mediated cell lysis. The complement system plays an important role bridging both innate and adaptive immune response to pathogens. The complement system can recognize and destroy pathogens based on PAMPs in addition to helping antibody–mediated lysis. The complement system is made of a cascade of proteins activated *via* three major pathways: the classical, alternative, and mannose–binding lectin pathway. The basic function of the complement system is to clear microbes and damaged cells from an organism, which promotes phagocytosis of particulate antigens, inflammatory responses, and immune clearance (67). HSV–1 and 2 evade complement–mediated destruction by expressing glycoprotein C, which binds to the C3b complement component, inhibiting both the classical and alternative complement pathways (68).

#### Autophagy

Autophagy is a cell death program activated when cells suffer nutrient starvation. During autophagy activation, cells digest their own cytoplasmic components and organelles in cytoplasmic lysosomes in order to recycle and scavenge various chemical species that may prolong their survival. Host cells can clear cytosol invading pathogens (viruses, bacteria, and protozoa) *via* autophagic degradation (69). Autophagy is important in viral antigen processing and presentation, mediating MHC class I or II presentation during the adaptive immune response. Selective viral autophagy plays a crucial role in antiviral host defense, for example, HSV–1 neurovirulence protein ICP34.5 binds the mammalian autophagy protein Beclin 1, inhibiting Beclin–1 dependent autophagy, as an innate immunity evasion mechanism (70).

#### Immune Evasion Strategies

Immune evasion is essential for the acute and chronic phases of herpesviruses infection (71). Viruses can encode for cytokine receptor genes acquired by the viral genome from the host to bind cytokines with high affinity and block their inflammatory response activity (72–75). Because less is known about innate immunity than adaptive immunity, understanding how herpesviruses manipulate mechanisms of innate immunity, as we describe below, can impact the development of improved therapeutic management of viral infections in order to prevent long term disorders and pathology of the central nervous system.

## IFN–Mediated Immune Response During HSV–1 Infection

The interferon response is induced during HSV–1 infection when PRRs in epithelial cells sense HSV–1 associated PAMPs (e.g., viral particles or viral replication products) (76). When IFNs are produced, they bind to their cognate receptors and activate IFN signaling cascades, resulting in the induction of IFN–stimulated genes (ISGs). ISG products create an antiviral state in the infected cells and neighboring uninfected cells to control the infection (77, 78).

Human myxovirus resistance protein B (MxB), an ISG product, is shown to restrict HSV–1 infection by inhibiting the delivery of incoming HSV–1 DNA to the nucleus, which is specified by its amino terminus and requires GTPase function (79). Human myxovirus resistance protein 1 (MxA) is an IFN–α/β induced antiviral protein that also inhibits replication of HSV–1, however the antiviral mechanism is not fully understood. A variant MxA (varMxA) isoform stimulated by HSV–1 infected cells in the absence of IFN–α induction enhanced production of infectious virus progeny in HSV‐1 infected cells (80). The VarMxA protein is expressed as a smaller 56 kDa variant and is alternatively spliced in HSV‐1‐infected cells. In contrast to IFN‐induced human MxA, which remains cytoplasmic, the varMxA protein is translocated into the nuclei of infected cells where it is associated with viral replication compartments and virions. This suggests that humans code for two MxA isoforms which is produced from alternative splicing (80, 81).

Three major classes of IFNs: IFN–1, IFN–2, and IFN–3 have been elucidated. These classes of IFNs compose the systematic response generated to combat HSV–1 infection. IFN–I limits the replication, spread, and cytopathic effect of HSV–1 (82–84). Studies show that increased viral replication, severe pathogenesis, and reduced survival rates are observed in mice lacking interferon–alpha/beta receptors (IFNAR) compared to WT controls (85, 86). IFN regulatory factor 3 (IRF3) and IFN regulatory factor 7 (IRF7), factors required for the induction of IFN–I production, are also both critical in controlling HSV–1 infection. Humans with IRF3 deficiencies are shown to be associated with Herpes simplex encephalitis (HSE) (87). An additional regulatory factor, IFN regulatory factor 1, is known to bind to the promoter of IFNβ and induce IFN–I response (88). To combat IFN–I response, microRNA–373 targets IRF1 which results in the suppression of ISG expression and promotion of HSV–1 replication. This suggests that HSV–1 can hijack the most miRNAs to promote replication by negatively regulating IFN–I production (89).

The IFN–II (i.e. IFNγ, or IFN–gamma) signaling pathway plays crucial roles in controlling and minimizing the pathogenesis of HSV–1 lytic infection (90). Mice lacking interferon–gamma receptors (IFNGR) were more susceptible to HSV–1 infection and had a higher mortality rate than WT mice (91–93). Furthermore, mice lacking both IFNGR and IFNAR had increased susceptibility to HSV–1 infection compared to mice lacking a single receptor (86). IFNγ can also directly inhibit the replication of HSV–1 through synergizing with IFNα and IFNβ (84, 94). IFNγ is also known to link the host innate and adaptive immune responses through stimulating the expression of major histocompatibility complex class I to enhance antigen presentation to CD8+ T cells. This linkage plays a key role in the maintenance of viral latency (92).

IFN–III (i.e. IFNλ, or IFN–lambda) utilizes the same signaling cascade as IFN–I. Studies have addressed the role of IFNλ during HSV–1 infection (95, 96). IFN–λ rapidly primes an IFN–I antiviral response in HSV–1–infected plasmacytoid dendritic cells (97). pDCs producing IFNλ during HSV–1 infection show a more efficient antiviral response in comparison to cells that don’t produce IFNλ (97). The underlying mechanism(s) of IFN–III during HSV–1 infection has yet to be elucidated.

### IFN–Mediated Immune Response During HSV–1 Infection in Neuronal Cells

Neuronal IFN signaling and its role in controlling acute and latent HSV–1 infection has recently been investigated. Neuronal antiviral response to HSV–1 is driven by IFN–β signaling (98). Sensory neurons respond to IFN–β, which then stimulates innate immunity and inhibits viral spread (99). However, multiple IFN types are involved in stimulating innate immunity. IFN–λ inhibits HSV–1 replication and viral protein synthesis in primary human astrocytes and neurons when exogenously treated (100).

HSV–1 replication is dependent upon autophagy. Specifically, HSV–1 is known to use the host endosomal sorting complexes required for transport (ESCRT) machinery for viral production and transportation (101, 102). As a defense mechanism, IFN–β and IFN–λ interfere with neuronal autophagy by subverting vacuolar protein sorting 4 (Vps4), a key protein involved in the ESCRT pathway. This is observed *in vivo* and in primary neurons where HSV–1 infection causes a decrease in Vps4 RNA and protein (103). Sensory ganglia also shows an accumulation of IFN–dependent LC3–decorated autophagic structures (LCS clusters) in result to HSV–1 infection (104). LC3 clusters appear to be associated with a delay in autophagy maturation, and resemble accumulations of autophagosomes and oversized autolysosomes *in vivo* (105).

IFN–β treatment in primary neurons and in other cell types is sufficient to transiently decrease Vsp4 RNA and protein levels. However, combined IFN–β and IFN–λ treatment recapitulate sustained LC3 clustering observed *in vivo*. Neighboring HSV–1 antigen–negative neurons also have decreased Vsp4 RNA and protein expression. It is speculated these neighboring neurons may be receiving IFN paracrine signaling, resulting in Vps4 reduction (105). Although HSV–1 downregulates IFN response and establishes lifelong latent infection in sensory neurons of the host, many studies show IFN response is critical for controlling HSV–1infection in neuronal and non–neuronal cells.

## Toll–Like Receptor (TLR) Signaling and HSV–1 Infection

**Figure 3:**
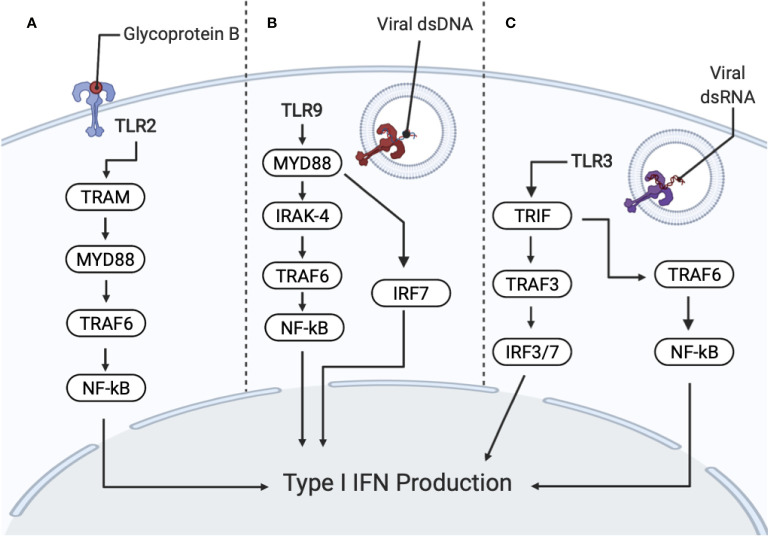
Recognition of HSV–1 by PRRs (pattern recognition receptors) activates interferon (IFN) and cytokine production. PRRs include toll–like receptors TLR2, TLR9 and TLR3. **(A)** TLR2 recognizes HSV–1 glycoprotein B (gB), promoting NF–κB activation and the secretion of interleukin (IL)–8 through the MyD88/TRAF6–dependent signaling pathway. Subsequent degradation of I–κBα (an inhibitor of NF–κB) allows NF–κB to translocate to the nucleus. TLR2 also signals through TRIF–related adaptor molecule (TRAM) and MyD88 for IFN production. **(B)** TLR9 senses viral DNA, which contains unmethylated CpG motifs. TLR9 is dependent on IRAK–4 and MyD88–dependent pathways. MyD88 and TRAF6 resulting in the activation of the NF–κB pathway for downstream cytokine secretion. TLR9 activation also signals IRF7, which produces type I IFNs. **(C)** TLR3 recognizes dsRNA, which are produced during viral replication. TLR3 activates MyD88–independent signaling cascade through the Toll/IL1 receptor domain, containing adaptor inducing IFNβ (TRIF) and TRAF3, resulting in IRF3/7 translocating to the nucleus, for the production of type I IFNs. TLR3 also signals TRIF and TRAF6, resulting in NF–κB activation.

The major TLRs activated during HSV–1 recognition that lead to the production of IFNs are summarized in **Figure 3**. TLRs are critical in controlling HSV–1 replication and dissemination by mediating antiviral activities during acute and latent infection. When TLRs bind to HSV–1 proteins or viral nucleic acid, they activate the innate immune response by inducing the production of chemokines and proinflammatory cytokines. This is accomplished through the signaling pathways of nuclear factor kappa–light–chain–enhancer of activated B cells (NF–κB), or p38 mitogen–activated protein kinase (MAPK) and c–Jun NH2–terminal kinase (Jnk) activation of activator protein–1 (AP–1), a transcription factor (106, 107). TLR expression varies among cell types such as macrophages and dendritic cells. TLRs are expressed differentially in the epithelial cells in HSV–targeted oral, ocular and genital mucosa (108) as well as in the central nervous system (CNS) resident cells (109, 110). Other studies show human neuronal cells express TLR family members 1–10 and IFN‐α/β during HSV–1 infection (64). The following expands on the role of important TLRs expressed during HSV–1 infection, primarily in neuronal cells. Relevant TLRs are listed and described in **Table 2**.

**Table 2:** Toll–Like Receptors Activated During HSV–1 Infection.

Toll–like receptor	Cellular Location	PAMP	Associated Factor
TLR2	Cell surface	Glycoprotein B	MyD88, TRAF6
TLR3	Endosome	dsRNA	TRIF, TRAF6
TLR9	Endosome	dsDNA	MyD88, TRAF6

### TLR2

TLR2 is a plasma membrane receptor that recognizes HSV–1 glycoprotein B (gB), promoting NF–κB activation and the secretion of interleukin (IL)–8 through the MyD88/TRAF6–dependent signaling pathway as shown in **Figure 3** (111). Induction of the degradation of I–κBα (an inhibitor of NF–κB) is followed after NF–κB activation, which allows NF–κB to translocate to the nucleus. This leads to the expression of several pro–inflammatory cytokines and chemokines in several human and mouse cell types, including epithelial, immune, and neuronal cells (78, 112–115). TLR2 also induces the IL–15 gene in response to HSV–1 infection (116). Additionally, IL–15 with IL–21 elicits proliferation of naive and memory CD8+ T Cells which contributes to controlling virus replication and spread (117). TLR2 is also found on the cell surface of microglia and astrocytes in the CNS, indicating that TLR2 plays a role in CNS autoimmunity, neurodegeneration, and tissue injury (118, 119). TLR2 mediates the inflammatory cytokine response to HSV–1 infection. Furthermore, TLR2 deficient mice have a blunted cytokine and chemokine response to HSV–1 infection (112). Furthermore, TLR2 synergizes with TLR9, which together controls viral replication and dissemination to the CNS (112, 120–122). TLR2 activation is also required to reduce the viral load in trigeminal ganglia and the brain during HSV–1 infection (122, 123). In TLR2 knockout mice, neuronal CCL2 levels were decreased, in association with reduced macrophage recruitment into the enteric nervous system after intragastric HSV–1 infection (124). TLR2’s role in the production of cytokines results in viral containment in response to HSV–1 infection.

### TLR3

TLR3 is found in cell compartments of microglia, astrocytes, oligodendrocytes, and neurons (58, 125–128). During HSV–1 infection, TLR3 is important for an efficient antiviral response. TLR3 recognizes double–stranded RNA (dsRNA) and induces the expression of type 1 IFNs and inflammatory cytokines upon activation of MyD88–independent signaling cascade (97, 106, 129–131). TLR3 localizes in endosomes and is TRIF and TRAF3–dependent for downstream signaling (132) (see **Figure 3**). TLR3 also signals through TRIF and TRAF6 for NF–κB and IRF–3 activation (132). Multiple studies suggest that TLR3 has an important role against HSV–1 in the CNS, supporting a model that the TLR3 axis, consisting of UBC93B, TRIF, TRAF3 and TBK1, exerts protective immunity to HSV–1 in the CNS (133–136). Furthermore, patients with TLR3 deficiencies or mutations are more susceptible to developing HSE (134, 137–139). TLR3 activation in neuronal cells is associated with increased resistance to HSV–1 infection and an increase in the production of IFNs and strengthened response to IFNs (64, 128, 140, 141). These studies reinforce the central role of type I IFNs and TLR3 as necessary components to contain viruses within the CNS (141).

### TLR9

TLR9 is found in endosomes/vacuolar compartments of microglia, astrocytes, dendritic cells and other antigen presenting cells. TLR9 recognizes dsDNA containing un–methylated CpG motifs (58, 125, 142, 143). During HSV–1 infection, TLR9 mediates an early and rapid production of type I IFNs and cytokine secretion through an IRAK–4 and MyD88–dependent pathway as shown in **Figure 3** (144–147). Interaction between TLR9 and other TLRs seems to be essential when mounting an effective immune response to HSV–1. In mice, defense against HSV–1 appears to be concentrated primarily in the TG (148). If the immune response to HSV–1 in the TG fails, a weak immune response is then seen in the brain. In WT mice, increased expression of TLR9 and TLR2 is seen in the TG, but not in the brain. Increased TLR expression in the brain is only observed in TLR2 deficient mice. TLR9 deficient mice are unable to mount an effective immune response in either location and die, despite the expression of other TLRs in the TG and brain (148). This indicates that in mice, TLR9 is important in coordinating the innate immune response with other TLRs. Further research is necessary to determine if the same is true in humans. TLR9 is required for IFN–α production in plasmacytoid dendritic cells (149). Furthermore, HSV–1 infection in human neurons was shown to be suppressed by IFN–λ, which upregulates TLR9 expression and subsequent TLR9–mediated antiviral responses involving the transcription factor IRF7 (150). This result remains to be validated as IFN–λ has been shown to be secreted during HSV–1 infection in the vaginal mucosa, mainly by dendritic cells (151).

Interestingly, TLR9 also coordinates with DNA sensors other than TLRs. The cGAS–STING pathway is a cytosolic DNA sensor (specific details on the mechanism of cGAS–STING signaling is provided in the next section). Like TLR9, cGAS–STING is also expressed in pDCs. Signaling through both the TLR9 pathway and the cGAS–STING pathway results in the induction of IFNs. Without modulation, this overlapping activation of IFN production could potentially lead to overproduction of IFN, which can have negative consequences. Crosstalk between the cGAS–STING pathway and the TLR9 pathway has recently been elucidated. Specifically, activation of the cGAS–STING pathway results in inhibitory signals that dampen the IFN production by the TLR9 pathway (152). The modulation of the TLR9 pathway by cGAS–STING is thought to be facilitated by two signals: suppressor of cytokine signaling 1 (SOCS1) and SOCS3. However, more research is required to determine the exact identity of the signal molecule.

TLR9 is a complex DNA sensor. Although its essential role in IFN production in pDCs is well established, more research in elucidating the role that TLR9 plays in coordinating with other TLRs and DNA sensors to coordinate an effective innate immune response to HSV–1 is necessary.

## cGas–Sting Pathway and HSV–1 Infection

**Figure 4:**
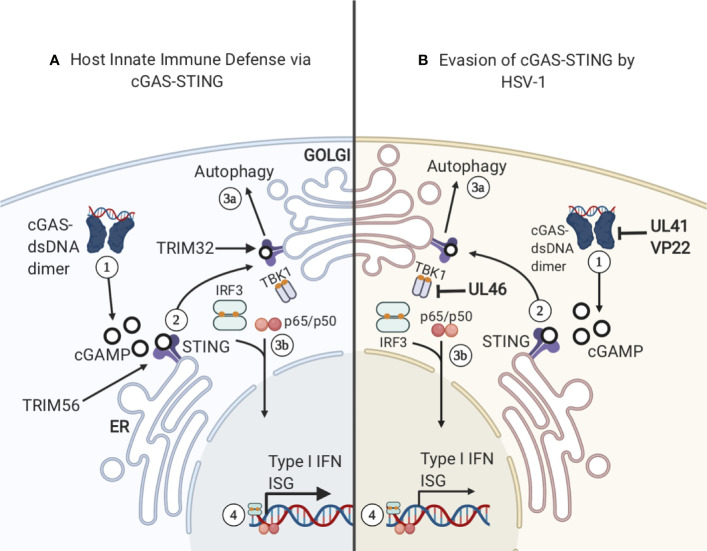
cGAS–STING. **(A)** 1) cGAS binds cytosolic dsDNA and they form a dimer. The cGAS–dsDNA dimer catalyzes the production of cGAMP, a ligand and secondary messenger for STING. 2) STING binds cGAMP and is ubiquitinated by TRIM56, inducing STING dimerization and translocation from the ER to the golgi. STING is then further ubiquitinated by TRIM32, allowing TBK1 to bind the complex. 3A) STING relocation to the golgi complex can activate autophagy in a pathway that is separate from the activation of IFN–related genes. 3B) TBK1 binding to STING triggers STING phosphorylation of S365 and the binding and activation of IRF3. Subsequently, IRF3 translocates to the nucleus and activates the transcription of IFN–1 and Interferon Stimulatory Genes (ISG). **(B)** HSV–1 has developed several mechanisms to sabotage cGAS-STING signaling. 1) Viral proteins UL41 and VP22 interact with cGAS, preventing the synthesis of cGAMP. 2) UL46 acts on TBK1 and prevents autophagy induction.

TLRs are an important mechanism for sensing HSV–1 and other viral infections. However, TLRs are not the only PRRs that can sense viral DNA. Another important PRR is cGAS–STING, a signaling pathway that detects cytosolic DNA and triggers a myriad of downstream immune responses. cGAS–STING has been the target of intense study as it has been identified as a potential universal cytoplasmic DNA sensor (153), and cGAS has also been implicated as the target of several strategies utilized by herpesviruses to evade the immune system (154). Due to the importance of cGAS–STING in responding to HSV–1 infection, the mechanism of cGAS-STING will be described further (see **Figure 4**). One of the outcomes of cGAS–STING signaling is the expression of Type I IFN genes, which help trigger the innate immune response. Upon cytosolic dsDNA detection, cGAS catalyzes the production of cyclic GMP–AMP (cGAMP), which serves as a second messenger and activator of STING (155). STING binding to cGAMP triggers ubiquitination of STING by TRIM56, inducing the dimerization of STING. The dimerized STING then translocates from the endoplasmic reticulum, where it usually resides, and moves to the golgi complex. Next, STING is poly–ubiquitinated by TRIM32, and serves as an anchor for the attachment of Tank binding kinase–1 (TBK1). Upon TBK1 binding to STING, TBK1 phosphorylates serine–365 (S365) on STING, facilitating the binding of IRF3 to STING. Subsequently, STING is phosphorylated by TBK1, leading to activation of IRF3 (154), a transcription factor whose activation leads to the transcription of IFN-1 Overall, phosphorylation of STING results in the induction of IFN-1.

### HSV–1 Mechanisms to Evade cGAS–STING Pathway

Coevolution of HSV–1 with humans has resulted in several mechanisms to bypass the immune response, resulting in HSV–1 circumventing or suppressing the cGAS–STING signaling pathway (see **Figure 4**). One mechanism is the expression of HSV–1 protein UL41, which reduces the expression of cGAS by degrading cGAS mRNA, and inhibits downstream activation of the IFN response (66) (**Table 3**). Additionally, HSV–1 protein VP22 interacts directly with cGAS and inhibits its enzymatic activity, preventing cGAMP production and STING activation (156). However, bypassing signaling by cGAS is not the only mechanism by which this pathway can be inhibited. HSV–1 protein UL46 has been shown to interfere with dimerization of TBK1, interfering with TBK1’s ability to interact with IRF3, ultimately resulting in a diminished IFN response (157). Interestingly, in addition to suppressing aspects of cGAS–STING signaling, there is some evidence that STING is required for optimal growth of HSV–1 (158). When HeLa cells were infected with a strain of HSV–1 lacking viral proteins ICP0 or ICP4, STING degradation was observed. These results suggest ICP0 and ICP4 are involved in stabilizing STING (158). Overall, STING has been shown to be both detrimental and required for HSV–1 replication (158). More research is needed to elucidate the exact relationship between STING degradation and HSV–1 replication.

**Table 3:** HSV–1 Proteins and Innate Immune Evasion.

Viral Protein	Action	Citation
UL41	Degrades cGAS mRNA	Su and Zheng (66)
VP22	Interacts directly with cGAS, suppressing cGAMP production	Huang et al. (156)
UL46	Inhibits dimerization TBK1	You et al. (157)

### IFN–Independent cGAS-STING Signaling

The myriad of mechanisms that HSV–1 has developed to bypass or inhibit DNA sensing *via* the cGAS–STING pathway indicates that this pathway is essential to coordinate an effective innate immune response. Interestingly, STING–deficient mice demonstrated an increased susceptibility to HSV–1 infection (159). Previously, it was assumed that increased susceptibility to HSV–1 infection resulting from cGAS–STING deficiency was solely the result of an impaired IFN response. However, recent work suggests that IFN production is one of multiple mechanisms triggered by cGAS–STING signaling pathway that combines to create an effective immune response (160). Mice with a serine 365–to–alanine mutation in STING, which renders STING unable to activate downstream IFN, demonstrates an increased resistance to HSV–1 infection when compared to mice with a STING–null phenotype (160). This raises the possibility that STING activation results in a series of IFN–independent signaling events that are also important in mounting an antiviral response. This activation does not involve S365, which is necessary for activating the IFN response, and instead relies on other STING domains. The evolutionary history of cGAS–STING supports this possibility. The cGAS–STING signaling pathway is demonstrably ancient, in fact, cGAS–STING homologs have been identified in the sea anemone *Nematostella vectensis*, which is divergent from humans by ~500 million years (161). Thus, it is possible that the role of STING in induction of IFN–based immunity is something that was taken up by the cGAS–STING pathway at a later point, as IFN–based immunity is likely a vertebrate evolutionary trait (162). More recent work indicates that the IFN–independent axis of cGAS–STING signaling contributes more to the immune response than previously thought (160). Although the exact details of this IFN–independent signaling pathway have yet to be fully elucidated, some evidence suggests that the function of the IFN–independent signaling pathway may be the induction of autophagy (163). A key event in autophagy induction is the conversion of the LC3 protein into its lipidated form, LC3–II, which takes place prior to the formation of autophagosomes (163). While LC3 lipidation can be induced by different mechanisms, cGAS production of cGAMP is sufficient to induce LC3 lipidation (163). When STING binds cGAMP, STING buds from the ER. After budding, STING interacts with protein transport protein SEC24C, allowing STING to bud into COP–II vesicles, forming the ERGIC complex (163). ERGIC acts as a locus for LC3 lipidation, leading to the formation of autophagosomes that clear cytosolic DNA or RNA (163). The involvement of STING in autophagosome formation supports the possibility that signaling through the cGAS–STING pathway also activates the autophagy response. Although this aspect of cGAS–STING signaling has only recently been elucidated in mammalian cells, autophagy induction is likely an ancient and evolutionarily conserved function of this pathway (163). The same motif for LC3 lipidation can be found in the STING homolog of *N. vectensis*, while the C–terminal domain which is essential for IFN signaling is absent (163). Taken together, these findings suggest that autophagy induction is indeed the ancient, evolutionarily conserved function of STING, and the induction of the IFN response was added in addition to the autophagy induction response.

### cGAS–STING Signaling in Non–Neuronal Cells

cGAS–STING is a key pathway in the CNS which senses and responds to HSV–1 infection (164). However, high viral load in the CNS produces an interesting phenotype in non–neuronal cells that is mediated by cGAS–STING signaling. More specifically, mice with herpes simplex encephalitis (HSE) exhibited increased apoptosis of microglia (brain–specific immune cells) (165). The apoptotic response appears to be independent of IFN–1 signaling, as IFNAR–deficient mice demonstrate an increased susceptibility to HSV–1, while not demonstrating less apoptosis of immune cells. In addition, apoptosis appears to be specific to microglia and other immune cells, as neurons and other neuronal cell types do not demonstrate the same degree of apoptosis as immune cells (165). Although this apoptotic response was initially observed in mice, apoptosis of immune cells was also observed in human organotypic cell culture and in tissue obtained from patients who had succumbed from HSE (165). The exact mechanism responsible for activation of apoptosis through cGAS–STING signaling is yet to be elucidated. It is thought that the apoptotic response in immune cells may function as a regulator of IFN–1 expression by the cGAS–STING signaling pathway (165). When the viral load during HSV–1 infection is low, local immune cells can produce IFN–1 *via* DNA sensing through cGAS–STING. However, prolonged expression of IFN–1 can lead to immunopathologies, especially in the brain, where prolonged inflammation can cause irreversible damage. To protect against damage from prolonged inflammation, it appears that cGAS–STING signaling is shut off by triggering local immune cells to initiate apoptosis, decreasing IFN–1 expression, despite elevated viral load (165). This represents a potential negative regulation of cGAS–STING signaling, and appears to be unique to non–neuronal cells, however more research is necessary to determine if this is truly unique to non–neuronal cells.

## Complement System and HSV–1 Infection

### Complement System and HSV–1 Infection of Non–Neuronal Cells

**Figure 5:**
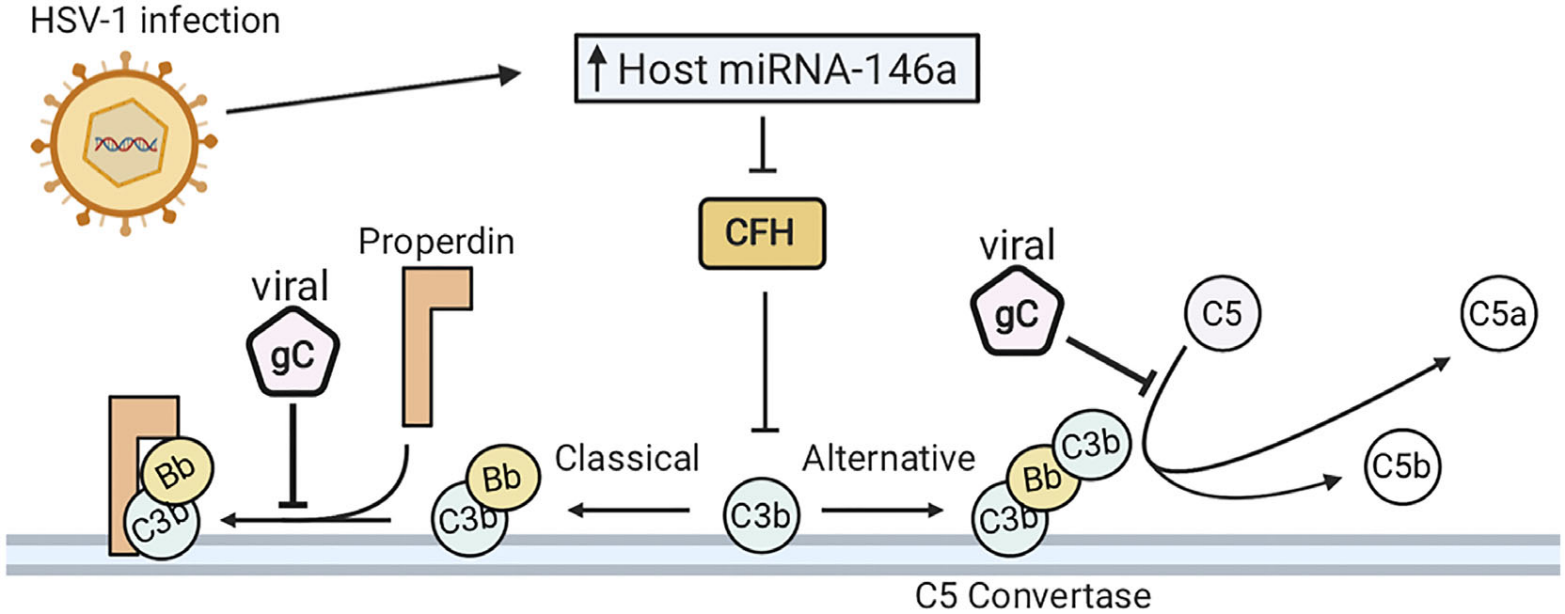
HSV–1 infection evades the complement system. (A) HSV–1 glycoprotein C (gC) binds complement component C3b. This inhibits the interaction of C5 and properdin with C3b, blocking activation of both the classical and alternative complement pathways. HSV–1 downregulates complement factor H (CFH), which is concurrent with elevated expression of host microRNA (miRNA) –146a.

HSV–1 has evolved multiple strategies to avoid immune evasion, many of which include inhibiting the complement system, whose proteins are found in serum and is part of the host innate immune response. The complement system is a cascade of proteins whose activation results in the formation of the membrane attack complex (MAC), a protein complex which penetrates the cell membranes of microbes by forming cytotoxic pores. In defense, HSV–1 encodes glycoprotein C (gC), a 511–amino–acid protein that plays several roles in host immune evasion (68, 166). More specifically, gC binds to the complement component C3b by interfering with the binding of C5 and properdin, thereby blocking alternative pathways that otherwise lead to the formation of a MAC on the pathogen surface, or the surface of virus–infected cells (see **Figure 5**) (167–169). Additionally, gC is able to accelerate the decay of the alternative pathway C3 convertase. Interestingly, HSV–1 lacking gC was more sensitive to complement–independent neutralization (170). These results suggest that HSV–1 gC is involved in immune invasion as it protects other viral envelope glycoproteins, including gB, which are essential for viral host cell entry and shielding these glycoproteins from neutralization as a potential mechanism of immune evasion (170).

### Complement System and HSV–1 Infection in Neuronal Cells

HSV–1 infection of human brain cells induces changes in gene expression, favorable to HSV–1 propagation and detrimental to the function of the host cells. Mechanistically, HSV–1 infection downregulates complement factor H (CFH), a complement regulator essential for controlling the complement pathway in blood and on cell surfaces (171, 172). When downregulated, CFH inhibits the amplification of the alternative pathway of complement activation (173). Downregulation of CFH is synchronous with elevated expression of host microRNA (miRNA) –146a (172). Furthermore, human primary neural cells infected with HSV–1 upregulate a brain–enriched miRNA–146a. Alterations in miR–146a expression levels can lead to pathogenesis of numerous neurological diseases. Furthermore, miRNA–146a is associated with proinflammatory signaling in stressed brain cells and Alzheimer’s Disease (AD) (174). HSV–1 DNA has also been detected in brain tissue from patients with AD (175–177). Overall, HSV–1 infection has developed strategies to evade the complement system in infected cells and human primary neural cells and can induce pathogenesis of AD.

## Conclusions and Future Perspectives

In this review, we summarized and discussed recent evidence of HSV–1 manipulating and evading host antiviral innate immune responses in both neuronal and non–neuronal cells. We described how HSV–1 induces IFN, TLR, and cGAS–STING mediated immune responses and overviewed mechanisms of how HSV–1 evades the innate immune response. The neuronal antiviral response to HSV–1 is driven by IFN signaling, which stimulates innate immunity and inhibits viral spread. The IFN response is critical for controlling HSV–1 infections in neuronal and non–neuronal cells as the IFN response establishes lifelong latent infection in sensory neurons of the host. Additionally, TLRs are critical in controlling HSV–1 replication and dissemination by mediating antiviral activities during acute and latent infection. TLRs bind to HSV–1 proteins or viral nucleic acid and activate the innate immune response by inducing the production of chemokines and proinflammatory cytokines. Previously, it was thought that cGAS–STING signaling only had IFN–1 expression as its most important function. However, new evidence indicates that cGAS–STING signaling also activates non–IFN related responses which are also important to mounting an effective immune response. Because cGAS–STING signaling is an important mechanism for controlling HSV–1 infection, HSV–1 has developed many ways to sabotage this pathway. Additionally, we reviewed the strategies HSV–1 utilizes to evade the complement system in infected cells and human primary neural cells, which can induce pathogenesis of Alzheimer’s Disease.

Most of the world’s population is infected by at least one α–HV, with ~90% of the world’s population infected with HSV–1 or HSV–2, or both (178). After initial α–HV infection, the host immune response plays a crucial role in clearing α–HVs from primary epithelial cells. As a result, α–HVs undergo latency in host neuronal cells in order to avoid immune system detection. Therefore it is crucial to consider that most normal immune responses likely involve latent herpesvirus infection and the virus plays important roles in patient’s responses to subsequent infections and predispositions to neurodegenerative as well as other chronic diseases (71). Thus, understanding how α–HV manipulates mechanisms of immunity can have major impacts for the development of improved therapeutic management of viral infections and improved quality of life.

Furthermore, as we continue to learn more about how HSV–1 can infiltrate the CNS, we will better understand how this life–long infection can impact neurological diseases such as Herpes Simplex Encephalitis (HSE), Multiple Sclerosis (MS) and Alzheimer’s disease (AD). While rare, HSE occurs in an estimated one in 250,000 to 500,000 HSV–1 infected individuals and can be life–threatening. Interestingly, the vast majority of adult cases of HSE are caused by HSV–1 infection (53, 179, 180). Furthermore, HSV–1 has been detected in the brains of both MS and AD patients more frequently than healthy controls (181–183). While the direct mechanisms by which HSV–1 may be contributing to the development of these diseases is controversial, several recent studies have pointed to the immune response during HSV–1 infection in the brain as a critical factor (53). These studies are extremely important, especially given the high prevalence of HSV–1 infection in humans worldwide. Overall, understanding the immune response and evasion mechanisms involved in both acute and latent HSV–1 infection may illuminate potential therapeutic targets to prevent long term neurological pathology.

## Author Contributions

AV, LM, S-JB, TD, NS and ES conceptualized and drafted the manuscript, as well as created the figures and tables. AV, LM, S-JB, TD, NS and ES all reviewed and edited the manuscript. All authors contributed to the article and approved the submitted version.

## Funding

AV is supported by NIH MBRS–RISE: R25–GM059298. LM is supported by Genentech Foundation Scholarship at SFSU. TD is funded by SPU Faculty Research and Scholarship Grant. NS is funded by NIH NIGMS 1SC2GM135135–01. ES is funded by the California State University Program for Education and Research in Biotechnology (CSUPERB).

## Conflict of Interest

The authors declare that the research was conducted in the absence of any commercial or financial relationships that could be construed as a potential conflict of interest.
